# Microfluidic Platform Integrated with Carbon Nanofibers-Decorated Gold Nanoporous Sensing Device for Serum PSA Quantification

**DOI:** 10.3390/bios13030390

**Published:** 2023-03-16

**Authors:** Emiliano Felici, Matías D. Regiart, Sirley V. Pereira, Francisco G. Ortega, Lúcio Angnes, Germán A. Messina, Martín A. Fernández-Baldo

**Affiliations:** 1 Facultad de Química, Bioquímica y Farmacia, Instituto de Química de San Luis, INQUISAL (UNSL—CONICET), Universidad Nacional de San Luis, Chacabuco 917, San Luis D5700BWS, Argentina; 2 GENYO, Centre for Genomics and Oncological Research, Pfizer/University of Granada/Andalusian Regional Government PTS, Granada, Avenida de la Ilustración, 114, 18016 Granada, Spain; 3 IBS Granada, Institute of Biomedical Research, Avenida de Madrid 15, 18012 Granada, Spain; 4 UGC Cartuja, Distrito Sanitario Granada Metropolitano. Calle Joaquina Eguaras, 2, 18013 Granada, Spain; 5 Laboratório de Automação e Instrumentação Analítica, Department of Fundamental Chemistry, Institute of Chemistry, University of São Paulo, Av. Professor Lineu Prestes 748, São Paulo 05508-000, Brazil

**Keywords:** electrochemical, immunosensor, microfluidic, carbon nanofibers, gold nanoporous, cancer biomarker

## Abstract

Prostate cancer is a disease with a high incidence and mortality rate in men worldwide. Serum prostate-specific antigens (PSA) are the main circulating biomarker for this disease in clinical practices. In this work, we present a portable and reusable microfluidic device for PSA quantification. This device comprises a polymethyl methacrylate microfluidic platform coupled with electrochemical detection. The platinum working microelectrode was positioned in the outflow region of the microchannel and was modified with carbon nanofibers (CNF)-decorated gold nanoporous (GNP) structures by the dynamic hydrogen bubble template method, through the simultaneous electrodeposition of metal precursors in the presence of CNF. CNF/GNP structures exhibit attractive properties, such as a large surface to volume ratio, which increases the antibody’s immobilization capacity and the electroactive area. CNFs/GNP structures were characterized by scanning electron microscopy, energy dispersive spectrometry, and cyclic voltammetry. Anti-PSA antibodies and HRP were employed for the immune-electrochemical reaction. The detection limit for the device was 5 pg mL^−1^, with a linear range from 0.01 to 50 ng mL^−1^. The coefficients of variation within and between assays were lower than 4.40%, and 6.15%, respectively. Additionally, its clinical performance was tested in serum from 30 prostate cancer patients. This novel device was a sensitive, selective, portable, and reusable tool for the serological diagnosis and monitoring of prostate cancer.

## 1. Introduction

The design and construction of effective devices for the real-time assessment of biomarkers to diagnose and monitor diverse diseases has represented an important research topic in the last 10 years. In this sense, microfluidic-based detection systems offer unique advantages, such as improved sensitivity, minimal reagent requirements and waste production, reduced costs, and a short analysis time [1]. These platforms make it possible to obtain miniaturized and portable devices and maintain homogeneous reaction conditions due to the high surface-to-volume ratio [2]. Currently, the vast range of techniques and materials used to develop microfluidic devices allow the development of designs with specific characteristics to fulfill particular application requirements [3,4,5]. In addition, microfluidic systems can be coupled with different detection techniques, with optical and electrochemical detection being the most commonly used [6,7,8]. In particular, electrochemical transducers exhibit superior sensitivity, portability and simplicity. The combination of these superior properties of electrochemical transducers with microfluidic platforms has paved the way to develop integrated devices with a wide range of applications in medicine, biochemistry, agri-food safety, environment security and industry [9,10,11]. Electrochemical detection allows interesting one-step electrode modifications, such as the dynamic hydrogen bubble template (DHBT) method that generates uniform gold nanopore structures (GNP), producing a large increase in determination sensitivity. This technique involves the electrodeposition of gold precursors while H_2_ bubbles are generated, resulting in a porous gold structure with highly desirable properties [12,13].

In addition, the DHBT procedure allows the incorporation of different nanomaterials to enhance the electrochemical surface. In this sense, carbon nanofibers (CNFs) are attractive, owing to their excellent electrical conductivity, low background current, large surface area, and high porosity [14]. Moreover, compared to carbon nanotubes, CNFs exhibit superior chemical stability, and thermal conductivity [15,16]. The inclusion of CNFs/GNP structures offers a stable surface and a simple way to incorporate recognition elements, generating a biorecognition platform that grants device specificity [17]. These and other inherent features make gold porous materials widely used in the design of sensing devices.

Consequently, several publications report electrochemical systems that incorporate GNP structures with interesting applications. For instance, Bertotti and co-workers demonstrated the possibility of determining anti-*Plasmodium vivax* (MSP119) antibodies in serum samples using a microfluidic system in which GNP structures were generated on a gold working electrode in the presence of CNT, reaching detection limits of 15 ng mL^−1^ [12]. Another recent example is the determination of different analytes using electrodes modified with GNP structures. Messina and co-workers described the quantification of ethinylestradiol in water samples. In this case, the electrochemical sensor was based on an imprinted electrode modified with GNP structures and graphene [13]. Ansarinejad and co-workers reported the use of an electrochemical sensor to determine piroxicam and tramadol using a polypyrrole/CuO nanocomposite-modified nanoporous gold film (NPGF) electrode [18]. Dantas and co-workers described the construction of a disposable gold microelectrode array with a gold nanoporous structure. This device was used for the electrochemical detection of inorganic and organic species by square-wave anodic stripping voltammetry [19]. Compared to these systems, our microfluidic device presents a novel and promising composite for sensing. The presence of CNF in combination with GNP allows us to reach excellent detection limits and remarkable selectivity by incorporating monoclonal antibodies specific to PSA (serum prostate-specific antigen).

In this context, an immuno-microfluidic device with a CNF/GNP structure in association with a platinum microelectrode constitutes an attractive, sensitive, and specific device to determine low levels of prostate cancer (PC) biomarkers. This pathology is one of the most common cancer types suffered by men, with an increasing incidence and mortality rate worldwide [20,21]. The etiology of PC has not been completely elucidated, although several factors could be associated with it, including aging, family history, and genetic mutations [20,22,23].

The Gleason score establishes the proper risk assessment and treatment selection through tissue biopsy [24]. Other non-invasive procedures are used for diagnosing and monitoring PC patients, including the Ki67 score, image analysis, and urine and blood biomarkers determination [25]. PSA is one the most important biomarkers for PC. This biomarker is a glycoprotein expressed in normal and cancerous prostate tissue. Values of PSA greater than 4 ng mL^−1^ are considered as a strong indicator of PC [26]. Serum PSA levels can change with age as well as non-PC related causes, such as urinary tract infections and medication [27,28]. The gold standard technique for PC diagnosis is based on colorimetric immunoassays (ELISA kit) for PSA quantification in serum samples, specific digital imaging studies (transrectal ultrasound guided prostate biopsy) and clinical medical expertise [26]. However, conventional ELISA methods are complicated, time consuming, expensive and require trained personnel, which restricts their use outside the laboratory, and therefore their portability. In this sense, a microfluidic immunosensor device that operates with a small sample and reagent volumes and reduces medical costs can be very interesting as an analytical tool for PC diagnosis and prognosis.

In this work, we have developed an analytical methodology based on a portable and reusable device, which stems from an immune-microfluidic system coupled with an electrochemical system of detection. This device has been fabricated for PSA quantification and validated in serum samples of healthy and PC donors. On the central channel of the developed device, a CNFs/GNP nanostructured platinum microelectrode has been placed.

This CNF/GNP composite increases the surface area and enhances biocompatibility. Nanostructured electrodes were functionalized with monoclonal capture antibodies against PSA, and captured PSA was quantified by the HRP-labeled antibodies as a sandwich type immunoassay. The results of this study suggest that this methodology offers a sensitive and specific method to quantify PSA as a biomarker in PC.

## 2. Materials and Methods

### 2.1. Reagents and Instruments

HAuCl_4_, CNFs (graphitized (iron free) composed of conical platelets, D × L 100 nm × 20–200 μm), bovine serum albumin (BSA), 3-mercaptopropionic acid (MPA), N-(3-dimethylaminopropyl)-N-ethylcarbodiimide (ECD), and N-hydroxysuccinimide (NHS) were acquired from Sigma-Aldrich (St. Louis, MI, USA).

Phosphate buffer saline (PBS pH 7.00), catechol, hydrogen peroxide (H_2_O_2_), acetic acid, and sulfuric acid (H_2_SO_4_) were purchased from Merck (Darmstadt, Germany). Enzyme-linked immunosorbent assay (ELISA kit) for PSA quantification was acquired from Thermo Fisher Scientific (Waltham, MA, USA). Monoclonal PSA antibodies (C-19) and HRP-conjugated antibodies were obtained from Santa Cruz (TX, USA). Poly(methyl methacrylate (PMMA) was purchased from All Acrylic (Sao Paulo, Brazil). Aqueous solutions were prepared by using purified water from a Milli-Q system.

Amperometry and cyclic voltammetry (CV) measurements were performed using a PGSTAT128N potentiostat from Metrohm Autolab (Metrohm, Barendrecht, the Netherlands), with NOVA 2.1 electrochemical software. Electrochemical measurements were carried out using a three-electrode cell (Pt wire as the auxiliary and working electrode, and Ag wire as the pseudo-reference electrode). Pt (Ø 125 µm) and Ag (Ø 500 µm) wire were obtained from Puratronic^®^-Alfa Aesar (Thermo Fisher Scientific, Waltham, MA, USA). Morphology and elemental characterization were achieved by a scanning electron microscope (SEM) using a LEO 1450 VP, with the energy dispersive spectrometer (EDS) EDAX Genesis 2000 (Oxford, UK). A syringe pump was used to introduce the solutions in the microfluidic device at a 2 μL min^−1^ flow rate (Baby Bee Syringe Pump, Bioanalytical Systems, West Lafayette, IN, USA). Absorbance was measured using a Bio-Rad Benchmark microplate reader and a Beckman DU 520 general UV/VIS spectrophotometer (Tokyo, Japan). All pH measurements were made with an Orion Expandable Ion Analyzer Model EA 940 (Orion Research Inc., Cambridge, MA, USA).

### 2.2. Microfluidic Device Fabrication

**Figure 1:**
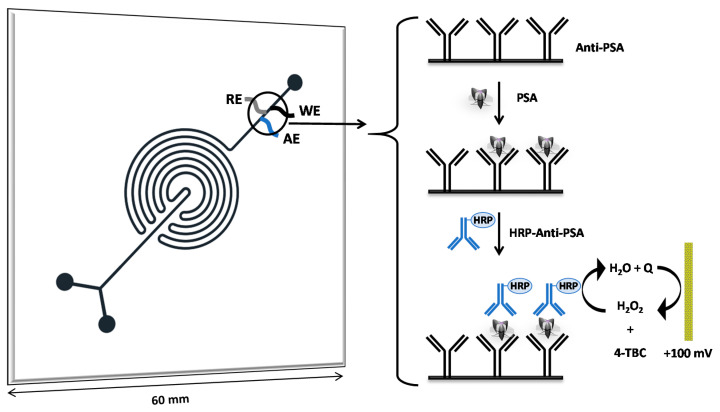
Representative scheme of microfluidic immunosensor device for PSA quantification.

The microfluidic device was designed using CorelDraw 12 software (Corel Corporation-version 12.0.0.458–2003) and transferred to the PMMA layer by CO_2_ laser-engraving (100 W laser machine Work Special 9060, from Visutec, Eisenstadt, Austria). The speed of the movement of the laser head and power parameters of the CO_2_ laser were optimized to achieve channels with the desired sizes (300 μm width and 150 μm depth). The labyrinthine configuration design consists of two inputs for reagents, samples and carriers, a chamber for the electrochemical cell, and an outlet for waste, as can be observed in Figure 1.

The microchannels engravings were made on a 6 cm × 6 cm × 0.5 cm PMMA plate. After that, drill holes (150 µm Ø and 525 µm Ø for Pt and Ag, respectively) were made in the chamber arranged for the electrochemical cell (Figure S1). Subsequently, the wires used as working and auxiliary (Pt 125 µm Ø × 3 mm length), and pseudo-reference (Ag 500 µm Ø × 3 mm length) electrodes were placed under pressure and sealed by superbonder glue. Finally, the PMMA plate was washed with Milli-Q water, dried, and thermally sealed onto a 6 cm × 6 cm × 2 mm PMMA plate in a heat press at 110 °C under 590 kPa for 45 min (Ferragini model HT3020, São Paulo, Brazil). The last step was the tube connection for the fluid external access from the syringe pump.

### 2.3. CNFs/GNP Electrode Modification

CNFs were previously pretreated in order to increase the dispersion according to the methodology described by Marin-Barroso and co-workers [29]. Later, CNFs/GNP structures were achieved by in situ co-electrodeposition on the Pt working electrode surface following the DHBT method. Firstly, 1 mL of a 50 μg mL^−1^ CNF dispersion was added to 1 mmol L^−1^ HAuCl_4_ in 0.5 mol L^−1^ H_2_SO_4_ solution and sonicated (50–60 Hz) for 15 min. Then, the dispersion was introduced to the microfluidic device and cycled, followed by applying a fixed −3 V potential for 150 s. At this potential, the GNP electrodeposition and the CNF reduction were achieved simultaneously [29]. Methodological conditions such as the electrodeposition time, electrodeposition potential and CNF concentration were optimized (Supplementary Material Figure S2). Finally, the CNFs/GNP-modified electrodes were washed with Milli-Q water several times, followed by SEM, EDS, and CV characterization.

### 2.4. Antibodies Immobilization

Firstly, 50 mmol L^−1^ MPA in EtOH:H_2_O (75:25, *v*/*v*) solution was cycled inside the microfluidic channel for 12 h at 25 °C. In this step, the MPA thiol group was covalently bound to the GNP surface, leaving free carboxylic groups, which were subsequently activated by 10 mmol L^−1^ EDC:NHS solution in PBS for 2 h at 25 °C. Moreover, the -COOH groups from the CNFs were also activated. Then, the microfluidic channels were washed with Milli-Q water several times and dried with N_2_.

Later, a 5 µg mL^−1^ anti-PSA monoclonal antibody solution in PBS was cycled for 12 h at 4 °C. Finally, the channels were washed with PBS several times, and stored in the same buffer at 4 °C. The microfluidic device was perfectly stable for at least 1 month.

### 2.5. Analytical Procedure for PSA Quantification

The microfluidic device, as well as the ELISA, were applied to the PSA determination in thirteen human serum samples with the aim of correlating both methodologies. Firstly, 1% BSA in PBS was introduced for 5 min as a blocking treatment to avoid non=specific bindings, followed by a PBS washing step for 5 min to eliminate the remaining material. After that, the human serum sample that was previously diluted 100-fold (following the ELISA protocol) was pumped for 5 min. In this step, the PSA antigen was specifically recognized by the anti-PSA monoclonal antibody, eliminating all other potential interferents in the sample matrix.

Then, following the sandwich-type immunoassay procedure, a secondary antibody labeled with horseradish peroxidase (HRP-anti-PSA) was injected for 5 min. Finally, 1 mmol L^−1^ catechol/H_2_O_2_ in 0.1 mol L^−1^ acetate buffer (pH 4.75) as the enzymatic substrate solution was introduced in the microfluidic device, and the quinone (enzymatic product) was detected at the CNFs/GNP structure at +100 mV.

In order to reuse the microfluidic device before the determination, 0.1 mol L^−1^ glycine pH 2 was used as a desorption solution, followed by a PBS washing step. In this step, the PSA antigen was desorbed from the anti-PSA antibodies, allowing us to perform a new determination step.

### 2.6. Serum Sample Collection

All patients gave written informed consent for the biological sample’s extraction to the Urology and Oncology Departments according to the Virgen de las Nieves University Hospital Ethical Committee and the Declaration of Helsinki principles. Blood samples were collected by puncture in a vacuum tube, followed by clotting without additives at room temperature for 30 min and centrifugation at 1500 g for 10 min. Finally, the supernatant was frozen at −80 °C until use.

### 2.7. Commercial ELISA Kit

ELISA determinations were performed according to the specific supplier’s instructions for PSA (Boston, MA, USA). The human-free PSA solid-phase sandwich ELISA kit is based on measuring the amount of this cancer biomarker bound between a matched antibody pair. A capture-specific antibody has been pre-coated in the wells of the supplied microplate. Samples, standards, or controls are then added into these wells and bind to the immobilized (capture) antibody. The sandwich is formed by the addition of the second antibody HRP and a substrate solution is added that reacts with the enzyme–antibody–PSA complex to produce a measurable signal. The intensity of this signal is directly proportional to the concentration of the PSA biomarker present in the serum sample. The measurement is photometrically taken at 450 nm.

## 3. Results and Discussion

### 3.1. CNFs/GNP Characterization

The CNF/GNP composite was synthesized via DHBT electrodeposition on the Pt working electrode. The DHBT electrodeposition method is based on the formation of H_2_ bubbles on the electrode surface by applying a negative potential in an acid medium. The H_2_ bubbles block the mass transport of Au ions to the nucleation sites on the electrode. Consequently, random micropores are formed during metal deposition. The honeycomb-like dendritic structure provides an improved rough surface area [30].

**Figure 2:**
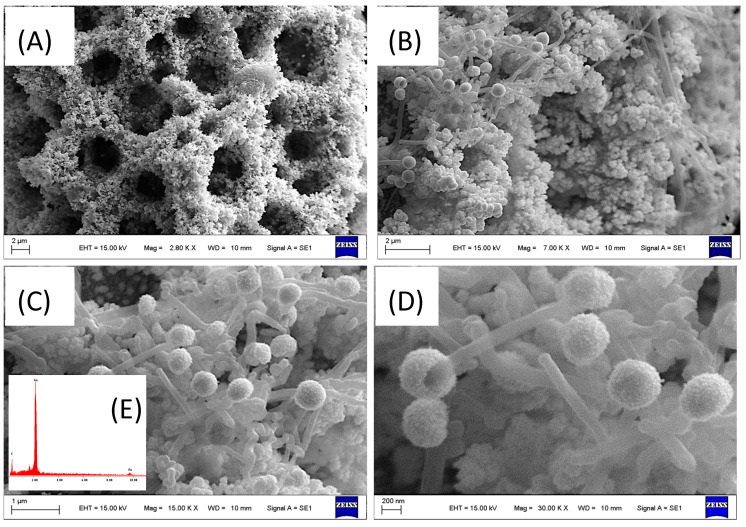
SEM micrographs of CNF/GNP composite at different magnifications. Inset: energy dispersive spectrum. (**A**) Uniform gold nanoporous honeycomb-like image. (**B**,**C**) GNP dendritic structure and the CNF interspersed in the gold by in situ co-deposition. (**D**) Electrode surface. (**E**) EDS spectrum.

The CNF/GNP composite was morphologically characterized by SEM at several magnifications. Figure 2A shows a characteristically uniform gold nanoporous honeycomb-like image. Moreover, at higher magnifications, the GNP dendritic structure and the CNF interspersed in the gold by in situ co-deposition (Figure 2B,C) are observed. The electrode surface shows structural defects because the carbon nanofibers form a network with the dendritic gold, as shown in a magnified image (Figure 2D). In addition, fungi-like formations with nanoporous gold spheres at the extremities of carbon nanofibers can be observed.

An EDS spectrum was analyzed to study the elemental composition. Figure 2E (Inset) shows the characteristic C (0.24 KeV) and Au (2.35, and 9.91 KeV) peaks. In the semi-quantitative microanalysis, the Au and C concentration was 86% and 14%, respectively.

**Figure 3:**
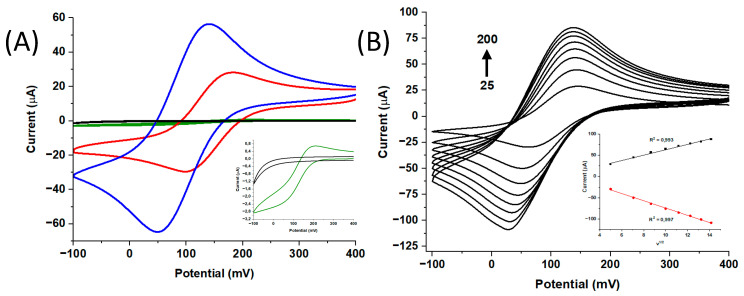
(**A**) CVs recorded in a 5 mmol L^−1^ [Fe(CN)_6_]^−3^ solution from +400 to −100 mV at 75 mV s^−1^ scan rate. Blank bare electrode measurement (black line), bare platinum microelectrode (green line), blank CNFs/GNP/Pt electrode measurement (pink line), GNP/Pt (red line), and CNFs/GNP/Pt (blue line). (**B**) Study of the scan rate influence on the peak current performed from 25 to 200 mV s^−1^ for the CNFs/GNP/Pt electrode in a 5 mmol L^−1^ [Fe(CN)_6_]^−3^ solution. Inset demonstrates the diffusion-controlled process proportionated by the CNFs/GNP/Pt microelectrode utilized in this study.

Cyclic voltammetry (CV) experiments were recorded in a 5 mmol L^−1^ [Fe(CN)_6_]^−3^ solution from +400 to −100 mV at a 75 mV s^−1^ scan rate. Figure 3A (inset) shows a characteristic sigmoidal curve for the bare platinum microelectrode (green line), in comparison with the blank bare electrode measurement (black line). Upon electrode modification, well-defined CV peaks corresponding to a [Fe(CN)_6_]^−3^ reversible redox process were recorded for the GNP/Pt and CNF/GNP/Pt measurement (Figure 3A), in comparison with the blank CNF/GNP/Pt electrode measurement (pink line). Moreover, an increase in current was observed for both modified electrodes compared to the bare electrode (green line). In addition, a shift in the potential towards less positive values was observed for CNFs/GNP/Pt (blue line) compared to GNP/Pt (red line). The first effect can be mainly attributed to the excellent electrical conductivity and the increased electroactive surface area. The second effect is due to the improvement in the electron transfer kinetics due to the greater number of active sites caused by the numerous defects generated by the carbon nanofibers interspersed in the gold dendritic porous surface.

In addition, the study of the scan rate influence on the peak current was performed for the CNF/GNP/Pt electrode in a 5 mmol L^−1^ [Fe(CN)_6_]^−3^ solution from +400 to −100 mV (Figure 3B). The experiments were carried out using a 25 to 200 mV s^−1^ scan rate. A linear relationship between both anodic and cathodic peak current values and the scan rate square root was observed, confirming that the [Fe(CN)_6_]^−3^ electrochemical behavior at the CNFs/GNP/Pt composite is a diffusion-controlled process.

### 3.2. Optimization of Experimental Parameters

Since the parameters of electrodeposition time (T_dep_) and potential (E_dep_) for the nanocomposite formation on the electrode surface were already optimized in a previous work [31], this work focused on the optimization of the experimental parameters for PSA quantification. To do this, a PSA standard solution of 10 ng mL^−1^ was used for all optimization experiments.

As the CNF concentration affects the electrochemical response, we evaluated the electrochemical signal in CNF concentrations ranging from 10 to 100 μg mL^−1^ (Figure S2A). It is worth noting that the current was significantly improved when the concentration increased up to 50 μg mL^−1^, reaching a plateau at higher concentrations. Therefore, we confirmed that the CNF concentration of 50 μg mL^−1^ was optimal for subsequent experiments.

Additionally, the concentration of the anti-PSA monoclonal antibody used in the immobilization procedure was optimized (Figure S2B). Several concentrations of the anti-PSA capturing antibody (1–10 µg mL^−1^) were covalently immobilized on the CNF/GNP nanostructure. After measuring the enzymatic reaction, we observed that the optimum antibody concentration was 5 µg mL^−1^.

Moreover, to determine the optimal pH for the enzymatic reaction (Figure S2C), the PSA measurement was tested in a pH range from 3.00 to 7.00. The obtained signal reached a maximum at pH 4.75, using acetate buffer as a solvent. The pH of the enzymatic reaction was then reached following buffer employment as the liquid of the reaction.

To find out the optimal flow rate (Figure S2D), several flow rates were evaluated while measuring the generated current during the immune reaction. Flow rates from 1 to 2.5 μL min^−1^ had little effect over the immune reaction. However, at a flow rate exceeding 3 μL min^−1^, the signal was dramatically reduced. Therefore, a flow rate of 2 μL min^−1^ was used for the sample, reagent and washing buffer injections.

### 3.3. Analytical Performance of the Electrochemical Device

**Figure 4:**
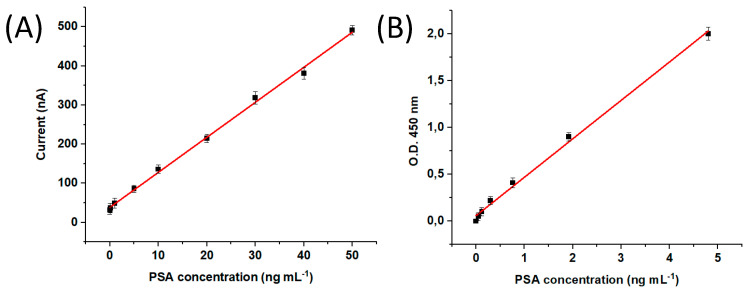
(**A**) Calibration curve of microfluidic immunosensor, and (**B**) calibration curve of commercial ELISA kit for PSA cancer biomarker.

The quantification of the PSA cancer biomarker was performed under the optimized parameters, and the results were analyzed in comparison with the commercial ELISA kit. The PSA calibration curve was constructed using 0.01 to 100 ng mL^−1^ standard solutions. A linear relationship was observed from 0.01 to 50 ng mL^−1^, according to the I (nA) = 38.57 + 8.95 C_PSA_ linear regression equation, with a R = 0.998 (Figure 4A). The commercial ELISA kit showed a linear relationship from 0.05 to 5 ng mL^−1^, according to the A (O.D.) = 0.05 + 0.41 C_PSA_ equation with a R = 0.996 (Figure 4B). The coefficients of variation (CV%) for the 10 ng mL^−1^ PSA standard solution were 3.85% and 6.45% (*n* = 5), and the limits of detection (LOD) were 5 and 45 pg mL^−1^ for the microfluidic immunosensor and the ELISA, respectively (IUPAC recommendations).

**Figure 5:**
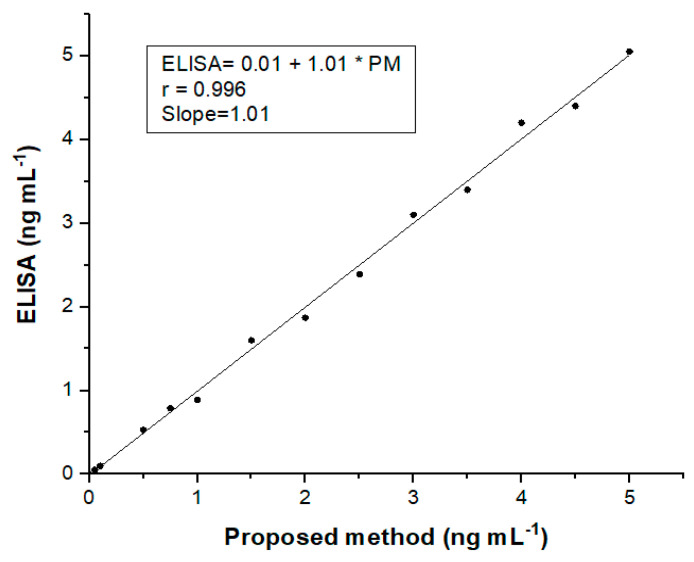
Correlation plot between the results obtained with the microfluidic immunosensor and the commercial ELISA kit for PSA when serial dilutions of PSA were analyzed.

Additionally, the correlation between both techniques was evaluated in several PSA dilutions. An excellent correlation between both methods was observed, which was indicated by the 1.01 straight line (Figure 5).

Moreover, the microfluidic immunosensor precision was evaluated by using the 10 ng mL^−1^ PSA standard. The within-assay precision was confirmed by five measurements on the same day. In the between-assay precision, the analyses were repeated for three consecutive days using different microfluidic devices. CV within-assay and between-assay values were below 4.40% and 6.15%, and 7.20% and 8.23% for the microfluidic immunosensor and the commercial ELISA kit, respectively. As can be observed in Table 1, the commercial ELISA kit requires 270 min for the analysis time, against the 21 min required by the proposed microfluidic sensor.

In addition, the stability of the sensor was investigated. For this purpose, the microfluidic immunosensor was stored at 4 °C in PBS for one month. A less than 5% loss of sensitivity was observed after storage compared to the response immediately after fabrication. The microfluidic immunosensor can be used for 20 days without a significant loss of sensitivity and allowed to perform about 15 serum sample analyses in a working day.

In addition, the selectivity against other possible cancer biomarkers in serum samples (EPCAM, EGFR, CEA and CA 15-3) was evaluated in 10-fold concentrations compared to PSA. The presence of these potential interference compounds caused less than 2% changes in the PSA quantification. The strong specificity was attributed to the blocking of non-specific adsorption (BSA) and the anti-PSA monoclonal antibodies. Finally, the developed immunosensor was tested in negative and positive control serum samples from PC patients and compared with the commercial ELISA kit as the gold standard assay (Table 2). The negative control samples were spiked with PSA in order to study the recovery percentage.

In comparison with a previous work [32], our microfluidic immunosensor based on the CNF/GNP nanocomposite platform for antibody monoclonal immobilization presents relevant advantages, such as its high surface and biocompatibility, miniaturization and easy handling, low-cost production, and short time analysis. In another recently published work [33], we developed an amperometric microfluidic immunosensor for claudin7 cancer biomarker determination in circulating extracellular vesicles (EVs) in colorectal cancer patient’s samples. Claudin7 is a relevant biomarker for colorectal cancer diagnosis and prognosis. The glass immunosensor consisted of a T-format with a central channel (60 mm length; 100 μm diameter) and side channels (15 mm length; 70 μm diameter). The sensor was based on synthesized MIL-125-NH_2_ particles (Materials Institute Lavoisier, titanium-oxo clusters and 2-aminoterephtalic acid linker) covalently anchored in the central channel. This nanomaterial was used as an efficient platform for the monoclonal antibody immobilization to recognize and capture this biomarker in EV samples. As an added value of this sensor compared to previous reports, this nanocomposite in the solid reaction phase is easier to use in the microfluidic device compared with magnetic nanoparticles, since there is no need for an external magnet. Interestingly, the modifications of the electrode’s surface did not reduce specificity in all the analyzed samples, showing a perfect correlation against the ELISA analyses, which indicates high versatility regarding the different kinds of samples. Further examples include the work of Takita and co-workers [34], who developed an aptasensor for PC diagnosis. This method was based on an electrochemical sensor combined with redox-labelled aptamers for PCA3 biomarker detection. This biomarker is overexpressed in PC patients’ urine. The detection mechanism consists of the increase in the charge transfer between the redox label and the electrode. This phenomenon is due to the aptamers recognized by the PCA3 proteins, bringing closer the redox labels (methylene blue) to the electrode surface.

In addition to the previously described articles, the design and construction of different sensors for PSA determination in serum samples have been reported. The main analytical features of these are summarized in Table 3. In this regard, it is essential to highlight that devices based on microfluidic systems allow the different steps of automation, reducing the determination process complexity. Moreover, our device’s reusability, portability, and short analysis time (21 min) facilitate the in situ PSA determination of multiple samples. Regarding the electrode modification, the carbon nanofibers (CNF)-decorated gold nanoporous (GNP) structures on Pt microelectrodes obtained by the dynamic hydrogen bubble template method represent a novel and simple strategy for constructing a selective immunoplatform. Finally, as observed in Table 3, our microfluidic device reached an adequate LOD that allows the detection of a PSA level that is clinically considered as an indicator of prostate cancer in serum samples.

## 4. Conclusions

We present a microfluidic immunosensor coupled with electrochemical detection based on a novel CNF/GNP nanocomposite platform for specific monoclonal antibody anti-PSA immobilization. This sensor was applied to the quantification of PSA biomarkers in serum samples. The analytical parameters such as linear range, precision and LOD, as well as the overall assay time required (21 min), were significantly improved according to the commercial ELISA kit (270 min) frequently used in clinical diagnosis. This sensor was tested using PC patients’ serum samples and validated against a commercial ELISA kit, showing an excellent correlation between both methods. The use of specific monoclonal antibodies as recognition biomolecules avoids potential cross reactivity in such a complex matrix and can be successfully applied to PSA detection in real human serum samples with high accuracy. Finally, our electrochemical method provides a truthful and useful analytical tool that can be easily used for PC diagnosis and prognosis in combination with digital rectal examination and imaging studies.

## Figures and Tables

**Table 1:** Comparison of the analytical performance between the commercial ELISA kit and the microfluidic immunosensor for PSA cancer biomarker.

Method	Time (min)	CV % ^a^ Within-Assay	CV % ^a^ Between-Assay	CV% ^a^	Linear Range	LOD
ELISA	270	7.20	8.23	6.45	0.05–5 ^b^	45 ^c^
MI ^d^	21	4.40	6.15	3.85	0.01–50 ^b^	5 ^c^

^a^ Five replicates (*n* = 5). ^b^ ng mL^−1^ PSA. ^c^ pg mL^−1^ PSA. ^d^ Microfluidic electrochemical immunosensor.

**Table 2:** Comparison of PSA data between the microfluidic electrochemical immunosensor and commercial ELISA kit for negative control (spiked) and positive control serum samples.

Samples	Addition ^b^	ELISA	Recovery%	MI ^d^	Recovery%
− ^a^	0	0	-	0	-
−	0.1	0.089 + 0.002 ^c^	89	0.097 + 0.001	97
−	1	1.11 + 0.04	111	1.04 + 0.02	104
−	5	4.91 + 0.07	98.2	5.04 + 0.04	100.8
−	10	9.71 + 0.09	97.1	9.95 + 0.06	99.5
−	25	24.1 + 0.15	96.4	25.3 + 0.07	101.2
−	50	47.7 + 0.21	95.4	49.7 + 0.09	99.4
+ ^e^	0	0.25 + 0.02	-	0.25 + 0.01	-
+	0	0.98 + 0.04	-	0.97 + 0.03	-
+	0	1.63 + 0.08	-	1.65 + 0.04	-
+	0	3.45 + 0.11	-	3.43+ 0.06	-
+	0	5.15 + 0.16	-	5.18 + 0.09	-

^a^ Control negative samples. ^b^ PSA spiked samples (ng mL^−1^). ^c^ Five replicates (*n* = 5) + SD. ^d^ Microfluidic electrochemical immunosensor. ^e^ Positive samples.

**Table 3:** Comparison of main analytical features of different immunosensors for PSA serum sample determination reported in the literature.

Assay Type	Platform	Detection Technique	Dynamic Range	LOD	Ref
Immunomagnetic assay	Microfluidic device	Amperometry	10 pg mL^−1^ to 1500 pg mL^−1^	2 pg mL^−1^	[32]
Label-free immunosensing	Ab/Ag2S/BiOBr/AgBr/Ag/ITO	Photo-electrochemistry	0.001 to 50 ng mL^−1^	0.25 pg mL^−1^	[35]
Label-free immunosensing	Ab/mucilage-GNPs-SNPs/GCE	DPV	0.1 pg mL^−1^ to 100 ng mL^−1^	0.078 pg mL^−1^	[36]
Label-free immunosensing	anti-PSA/AuNPs/PANI/MWCNTs-COOH/GCE	DPV	1.66 pg·mL^−1^ to 1.3 ng·mL^−1^	0.5 pg·mL^−1^	[37]
Sandwich-type immunoassay	Ab/Fe3O4/MWCNT/GCE	DPV	2.5 pg mL^−1^ to 100 ng mL^−1^	0.39 pg mL^−1^	[38]
Label-free immunosensing	Ab-Cs-rGO/AuNRs-FTO	DPV	0.1 to 150 ng mL^−1^	16 pg mL^−1^	[39]
Sandwich-type immunoassay	Ab/AuNPs-ATPGO/GCE and d-Ti3C2TX MXene@AuNPs as label of Ab_2_	DPV	0.01 to 1.0 pg mL^−1^	3 × 10^3^ pg mL^−1^	[40]
Sandwich-type immunoassay	Ab/CHIT-MOF/GCE and QDs as label of Ab_2_	DPV	1 pg mL^−1^ to 100 ng mL^−1^	0.45 pg mL^−1^	[41]
Label-free immunosensing	Ab/AuNPs/CS–GR–IL–Fc cry/SPCE	DPV	1.0 × 10^−7^ to 1.0 × 10^−1^ ng mL^−1^	4.8 × 10^−5^ pg mL^−1^	[42]
Sandwich-type immunoassay	Ab/AuNPs/GCE and MOF-235/MB as label of Ab_2_	DPV	10 to 1200 pg⋅mL^−1^	3 pg⋅mL^−1^	[43]
Sandwich-type immunoassay	Microfluidic device with Ab/CNFs/GNP/Pt electrode	Amperometry	0.01 to 50 ng mL^−1^	5 pg mL^−1^	This work

ITO: iridium tin oxide, PANI: polyaniline, Cs: chitosan, FTO: fluorine-doped tin oxide electrodes, ATPGO: ATP-functionalized graphene oxide, MXene: known as metal carbides/carbonitrides; d-Ti3C2TX MXene@AuNPs: delaminated MXene-gold nanoparticles, Ab_2_: secondary antibody, CS–GR–IL–Fc cry: 3D porous cryogel of chitosan, graphene, ionic liquid and ferrocene, MOF: metal organic framework, MB: methylene blue.

## Data Availability

The data presented in this study are available in the Supplementary Materials and on request from the corresponding author.

## Supplementary Materials

The following supporting information can be downloaded at: https://www.mdpi.com/article/10.3390/bios13030390/s1, Figure S1. Design of the electrochemical cell and microscopy photo; Figure S2. Optimization of Experimental Parameters. (A) CNF concentration, (B) PSA antibody concentration, (C) pH, and (D) Flow rate.
