# Supplementary material for: Microfluidic Platform Integrated with Carbon Nanofibers-Decorated Gold Nanoporous Sensing Device for Serum PSA Quantification
Source: Biosensors (Basel). 2023 Mar 16;13(3):390. doi: 10.3390/bios13030390 (PMC10046291; doi:10.3390/bios13030390)
Supplement: Supplementary file 1 [file biosensors-13-00390-s001.zip › biosensors-2216611-supplementary.pdf]

biosensors

MDPI

Supporting Information

Microfluidic Platform Integrated with Carbon Nanofibers-Decorated Gold Nanoporous Sensing Device for Serum PSA Quantification

Emiliano Felici [1], Matías D. Regiart [1,*], Sirley V. Pereira [1], Francisco G. Ortega [2,3,4], Lúcio Angnes [5], Germán A. Messina [1] and Martín A. Fernández-Baldo [1,*]

[1] Facultad de Química, Bioquímica y Farmacia, Instituto de Química de San Luis, INQUISAL (UNSL—CONICET), Universidad Nacional de San Luis, Chacabuco 917, San Luis D5700BWS, Argentina
[2] GENYO, Centre for Genomics and Oncological Research, Pfizer/University of Granada/Andalusian Regional Government PTS, Granada, Avenida de la Ilustración, 114, 18016 Granada, Spain
[3] IBS Granada, Institute of Biomedical Research, Avenida de Madrid 15, 18012 Granada, Spain
[4] UGC Cartuja, Distrito Sanitario Granada Metropolitano.Calle Joaquina Eguaras, 2, 18013 Granada, Spain
[5] Laboratório de Automação e Instrumentação Analítica, Department of Fundamental Chemistry, Institute of Chemistry, University of São Paulo, Av. Professor Lineu Prestes 748, São Paulo 05508-000, Brazil
* Correspondence: regiart@unsl.edu.ar (M.D.R.); mbaldo@unsl.edu.ar (M.A.F.-B.); Tel.: +54-266-4425385

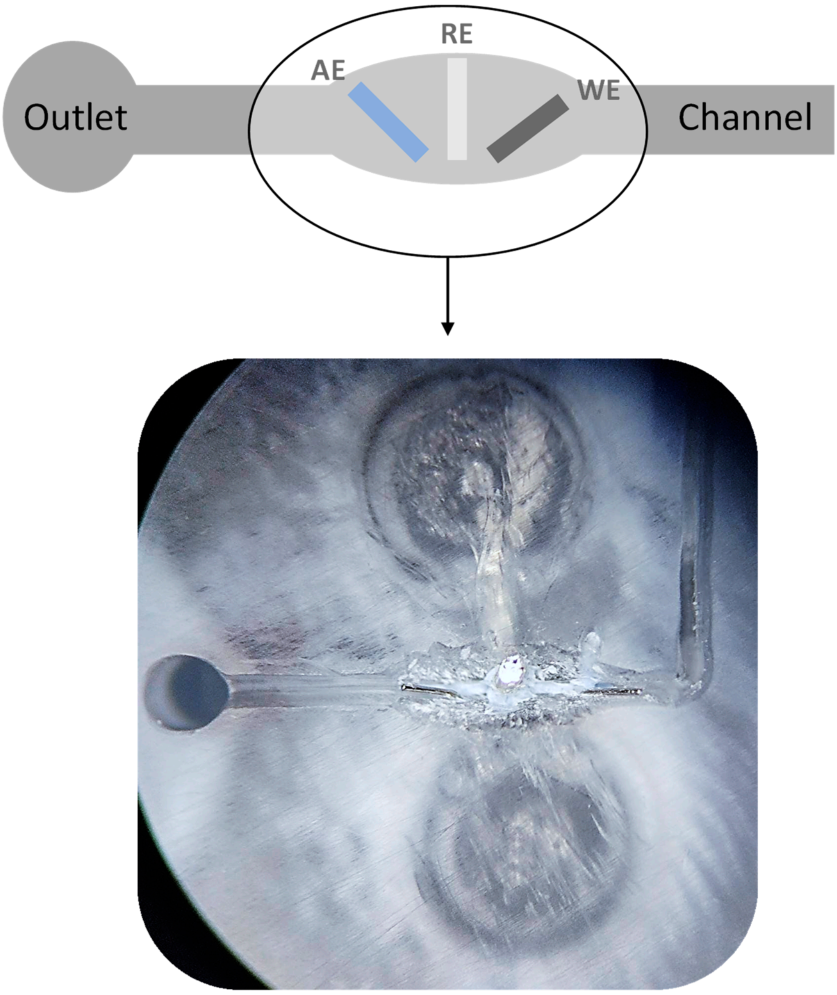

Figure S1. Design of the electrochemical cell and microscopy photo.

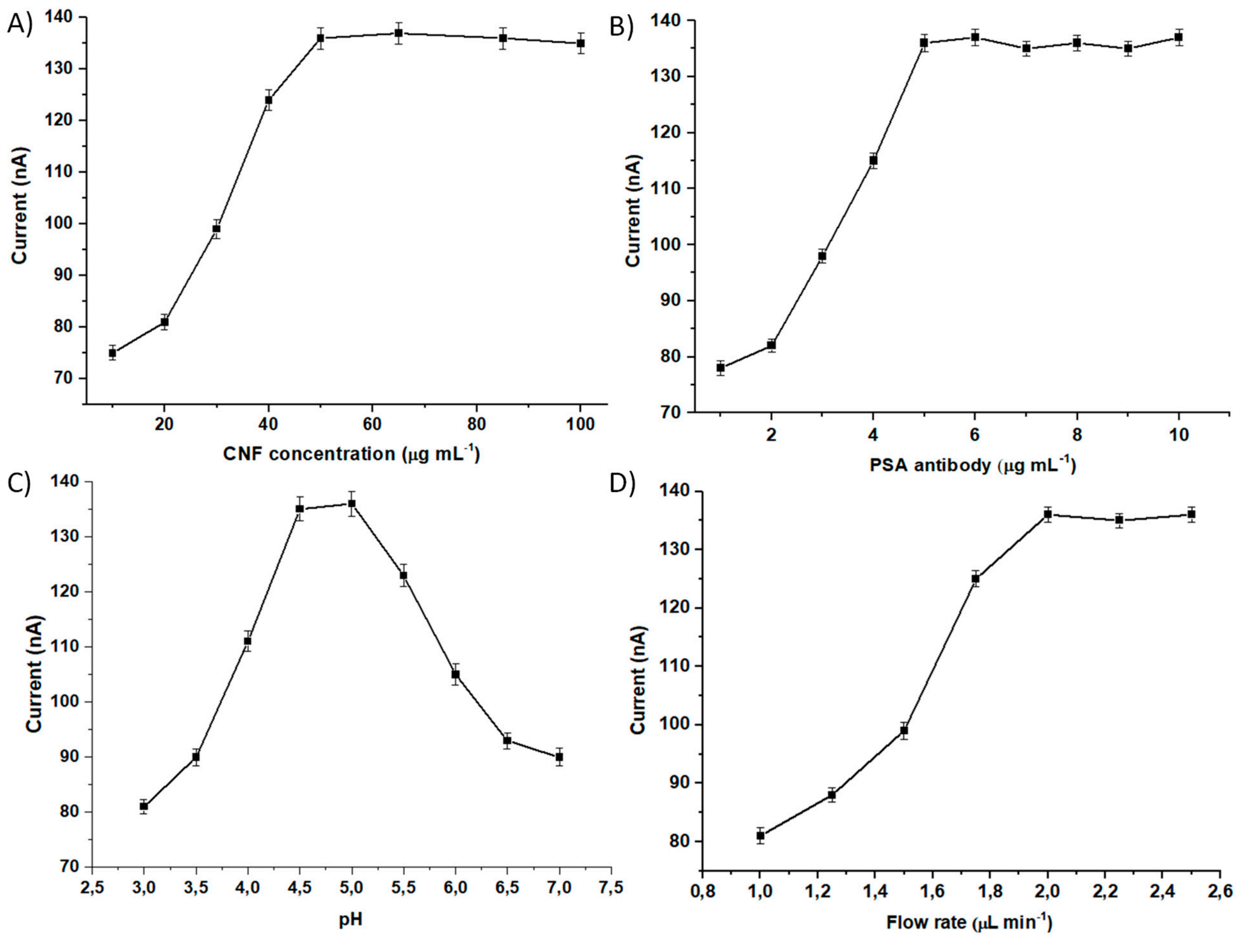

Figure S2. Optimization of Experimental Parameters. A) CNF concentration, B) PSA antibody concentration, C) pH, and D) Flow rate.
